# Supplementary material for: High-Efficiency Separation of Mg2+/Sr2+ through a NF Membrane under Electric Field
Source: Membranes (Basel). 2021 Dec 31;12(1):57. doi: 10.3390/membranes12010057 (PMC8781883; doi:10.3390/membranes12010057)
Supplement: Supplementary file 1 [file membranes-12-00057-s001.zip › membranes-1500889-supplementary.pdf]

membranes

Editorial Materials: High-Efficiency Separation of Mg^{2+}/Sr^{2+} through a NF Membrane under Electric Field

Huan Liu [1], Quan Li [1], Benqiao He [1,*], Zhengguang Sun [2], Feng Yan [3], Zhenyu Cui [1] and Jianxin Li [1]

1 State Key Laboratory of Separation membranes and Membrane Processes, School of Materials Science and Engineering, Tiangong University, Tianjin 300387, China; lh962854@163.com (H.L.); leequan92@163.com (Q.L.); cuizheyhh@163.com (Z.C.); jxli@tiangong.edu.cn (J.L.)
2 School of Materials Science & Engineering, Hubei University, Wuhan 430062, China; sunshine@hubu.edu.cn
3 School of Environmental Chemistry and Engineering, Tiangong University, Tianjin 300387, China; yanfeng@tiangong.edu.cn (F.Y.)
* Correspondence: hebenqiao@tiangong.edu.cn

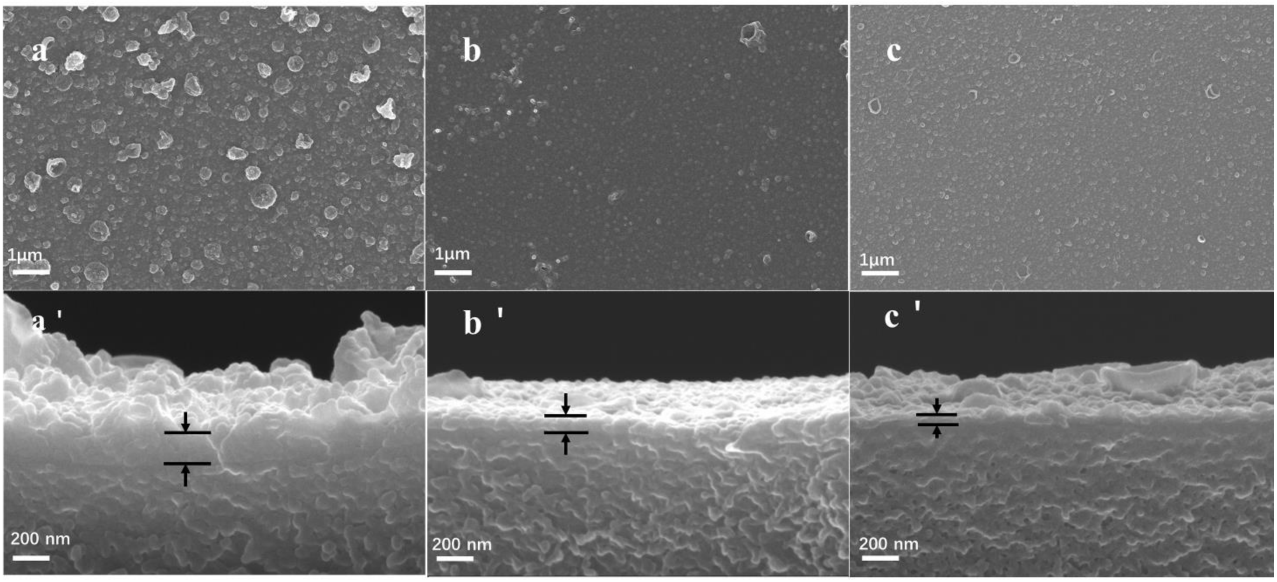

Figure S1. SEM images of; (**a**) NF1, (**b**) NF2 and (**c**) NF3.

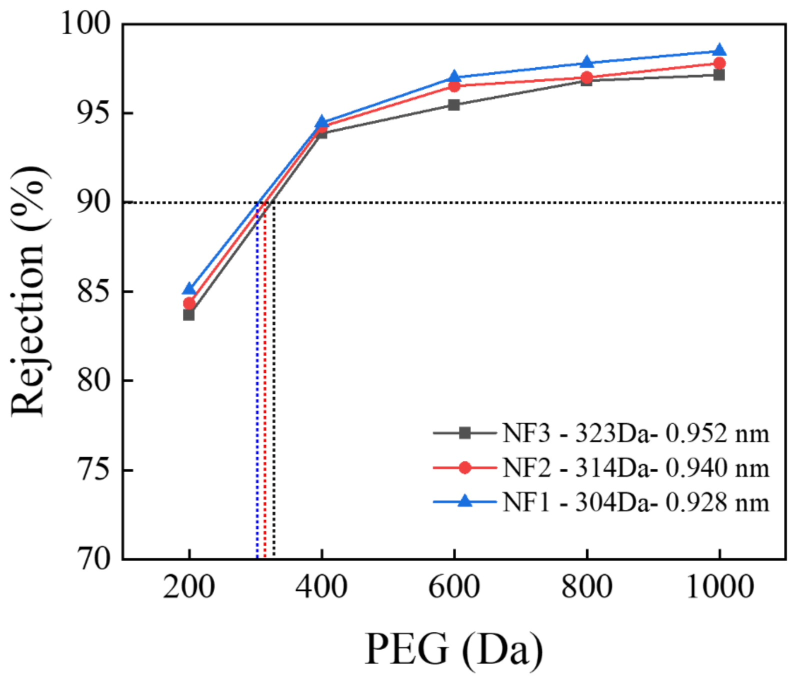

Figure S2. Pore sizes of NF membranes.

Table S1. Monomer concentrations used for NF membranes.

Membranes	PIP (wt.%)	TMC (wt.%)
NF1	2	0.5
NF2	1	0.25
NF3	0.5	0.125

Table S2. Ionic radii (r), hydrated radii (r′), Hydration free energy, and diffusion coefficients (D, infinite dilution at 25 °C) of common cations[1].

Ion	Ionic radius nm	Hydrated radius	Hydration free energy KJ/mol	Diffusion coefficients 10−9 m2 s−1
Li^+	0.076	0.340	-515	1.029
Na^+	0.098	0.276	-365	1.334
K^+	0.133	0.201	-271	1.957
Mg^{2+}	0.066	0.428	-1828	0.706
Ca^{2+}	0.100	0.412	-1504	0.792
Sr^{2+}	0.113	0.412	-1379	0.791

Reference

1. Tang, C.; Bruening, M.L.J.J.o.P.S. Ion separations with membranes. *Journal of Polymer Science* **2020**, *58*. https://doi.org/10.1002/pol.20200500.
